# Ugonin M, a *Helminthostachys zeylanica* Constituent, Prevents LPS-Induced Acute Lung Injury through TLR4-Mediated MAPK and NF-κB Signaling Pathways

**DOI:** 10.3390/molecules22040573

**Published:** 2017-04-01

**Authors:** Kun-Chang Wu, Shyh-Shyun Huang, Yueh-Hsiung Kuo, Yu-Ling Ho, Chang-Syun Yang, Yuan-Shiun Chang, Guan-Jhong Huang

**Affiliations:** 1 Department of Chinese Pharmaceutical Sciences and Chinese Medicine Resources, College of Chinese Medicine, China Medical University, Taichung 40402, Taiwan; johnwu0919@gmail.com (K.-C.W.); kuoyh@mail.cmu.edu.tw (Y.-H.K.); tim.tim0619@msa.hinet.net (C.-S.Y.); 2 School of Pharmacy, China Medical University, Taichung 40402, Taiwan; sshuang@mail.cmu.edu.tw; 3 Department of Biotechnology, Asia University, Taichung 41354, Taiwan; 4 Department of Nursing, Hungkuang University, Taichung 43302, Taiwan; elaine@sunrise.hk.edu.tw; 5 Chinese Crude Drug Pharmacy, China Medical University Hospital, Taichung 40402, Taiwan

**Keywords:** acute lung injury, *Helminthostachys zeylanica*, Ugonin M

## Abstract

*Helminthostachys zeylanica* (L.) Hook. is plant that has been used in traditional Chinese medicine for centuries for the treatment of inflammation, fever, pneumonia, and various disorders. The aims of the present study are to figure out the possible effectiveness of the component Ugonin M, a unique flavonoid isolated from *H. zeylanica*, and to elucidate the mechanism(s) by which it works in the LPS-induced ALI model. In this study, Ugonin M not only inhibited the production of pro-inflammatory mediators such as NO, TNF-α, IL-1β, and IL-6, as well as infiltrated cellular counts and protein content in the bronchoalveolar lavage fluid (BALF) of lipopolysaccharides (LPS)-induced acute lung injury (ALI) mice, but also ameliorated the severity of pulmonary edemas through the score of a histological examination and the ratio of wet to dry weight of lung. Moreover, Ugonin M was observed to significantly suppress LPS-stimulated protein levels of iNOS and COX-2. In addition, we found that Ugonin M not only obviously suppressed NF-κB and MAPK activation via the degradation of NF-κB and IκB-α as well as ERK and p38MAPK active phosphorylation but also inhibited the protein expression level of TLR4. Further, Ugonin M treatment also suppressed the protein levels of MPO and enhanced the protein expressions of HO-1 and antioxidant enzymes (SOD, GPx, and CAT) in lung tissue of LPS-induced ALI mice. It is anticipated that through our findings, there is strong evidence that Ugonin M may exert a potential effect against LPS-induced ALI mice. Hence, Ugonin M could be one of the major effective components of *H. zeylanica* in the treatment of inflammatory disorders.

## 1. Introduction

Acute lung injury (ALI) is a major clinical complication with high rates of morbidity and mortality, characterized by alveolar-capillary membrane damage [1]. In the most recent randomized trials, overall 28-day mortality was reported as 25%–30%, whereas mortality in community-based surveys was 35%–40% [2,3]. Research efforts targeting ALI treatment have focused primarily on the innate immune system, and have typically conceptually viewed ALI as a syndrome of hyper-inflammation [4]. Responding to inflammatory stimulation, the primary sources of cytokines in the lungs are macrophages, which play an important role in the pathogenesis of lung injuries [5].

TLR4, as a member of the Toll-like receptor family, is responsible for activating the innate immune system. It is most well-known for recognizing lipopolysaccharides (LPS), a component present in many Gram-negative bacteria and select Gram-positive bacteria (e.g. *Neisseria spp*) [6,7]. Upon LPS recognition, TLR4 results in oligomerization and recruits its downstream adaptors through interactions with the TIR (Toll-interleukin-1 receptor) domains, which is followed by the accumulation of TIR domain containing adaptor inducing IFN-β (TRIF), and subsequently the activation of the mitogen activated protein (MAP) kinase pathway, the nuclear factor kappa-light-chain-enhancer of activated B cells (NF-κB) family of transcription factors, and the IFN regulatory factor (IRF) family of transcription factors. This then results in the inflammatory production of interferon (IFN) and cytokines [4,8]. Therefore, blocking the TLR4-mediated signaling pathways may be a more effective approach to treating ALI [9].

Various plant extracts and their constituents showed therapeutic effects against lung inflammatory disorders, including coumarins, flavonoids, phenolics, iridoids, monoterpenes, diterpenes and triterpenoids. Some of them exerted inhibitory effects mainly by modulating the NF-κB and MAPK pathways. Especially, many flavonoid derivatives, such as Baicalin, Hesperidin, Luteolin, and Naringin, obviously presented effectiveness on lung inflammatory disorders [10].

The traditional Chinese medicine, the root and rhizoma of *Helminthostachys zeylanica* (*H. zeylanica*), known colloquially as “Ding-Di-U-Gon”, has been used for centuries in the treatment of inflammation, fever, pneumonia, burns, and various disorders. The plant has been shown to possess an array of medicinal properties [11,12]. Moreover, it has been demonstrated to exhibit antioxidants, anti-inflammatory, antipyretic, and hepatoprotective activities [13,14,15,16,17,18]. Chemical components of *H. zeylanica* contain stilbenes and flavonoids [13,19]. Ugonin M (C_25_H_24_O_7_) is a unique flavonoid isolated and identified in *H. zeylanica* [15]. Its chemical structure is shown in Figure 2A. One study has shown the anti-inflammatory value of Ugonin M in the inhibition of superoxide anion generation and elastase release [15]. Another study has shown that extract of *H. zeylanica* attenuates LPS-induced ALI in mice by modulating NF-κB and MAPK pathways [20]. However, the effective component of *H. zeylanica* and further mechanisms of Ugonin M in LPS-induced pulmonary inflammation remain unclear. Thus, the aims of the present study are to figure out whether Ugonin M is a possible effective component of *H. zeylanica* and to elucidate the mechanism(s) by which it works in an ideal model of LPS-induced ALI.

## 2. Results

### 2.1. HPLC Analysis of H. zeylanica

**Figure 1:**
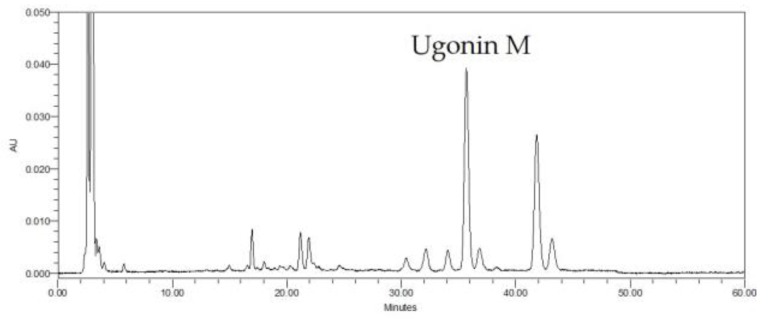
Chromatographic analysis of *H. zeylanica* detected at 360 nm. The major peak on the chromatography of the ethanol extract of *H. zeylanica* was identified as standard Ugonin M compound.

HPLC chromotogram was established for *H. zeylanica* (Figure 1). We found that Ugonin M was the major peak on the chromatography of the ethanol extract of *H. zeylanica* (retention time, 35.695 min). The maximum absorbance was selected at 360 nm.

### 2.2. Cytotoxicity and the Effect(s) of Ugonin M on NO Production in Raw 264.7 Cell

**Figure 2:**
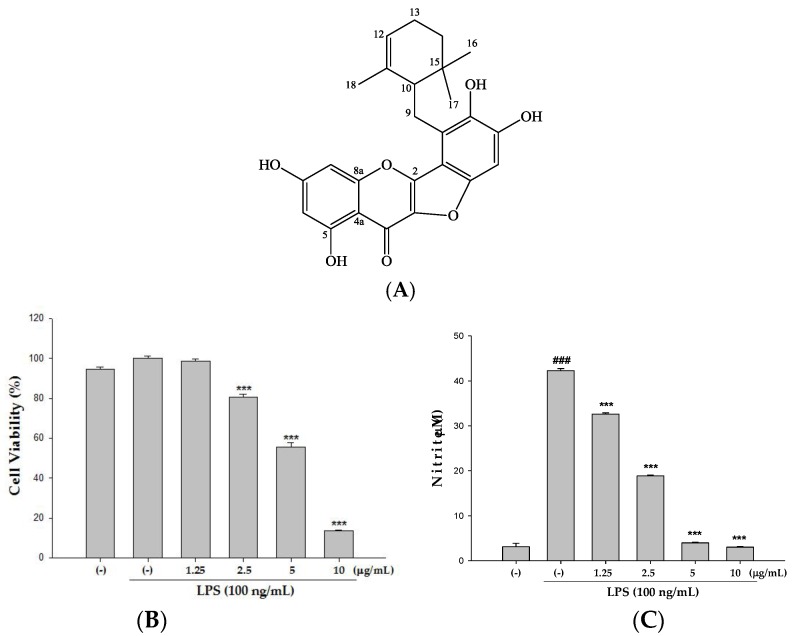
(**A**) Chemical structures of Ugonin M from *Helminthostachys zeylanica*; (**B**) cytotoxicity; and (**C**) effect of NO production of Ugonin M in lipopolysaccharides (LPS)-stimulated RAW264.7 cells. Cells were pretreated with different concentrations of Ugonin M, 10, 5, 2.5, 1.25, or 0 μg/mL (0 referred to as (−)), for 1 h prior to the addition of 100 ng/mL LPS for 24 h. Cell viability assay was performed using MTT assay. Nitrite concentration in the medium was determined using Griess reagent. The data were presented as mean ± SD for the three different experiments performed in triplicate. ^###^ *p* < 0.001 was compared with a sample of the control group (one-way ANOVA followed by Scheffe’s multiple range tests). *** *p* < 0.001 was compared with the LPS-only group.

Prior to the in vivo study, we first examined the cytotoxic effect of Ugonin M on Raw 264.7 cells using the MTT colorimetric assay. Raw 264.7 cells were treated with 1.25–10 μg/mL Ugonin M. LPS was added one hour after incubation. The results in Figure 2B show that LPS does not induce cell death, and the percentage of cytotoxicity induced by Ugonin M within the range of 1.25–2.5 μg/mL is lower than 20%. As a result, in all subsequent animal experiments, only doses below or equal to 2.5 μg/mL were applied. To check the effect(s) of Ugonin M on LPS-induced NO production, RAW 264.7 cells were treated with a variety of concentrations (1.25–10 μg/mL) of Ugonin M and LPS (100 ng/mL) for 24 h. As shown in Figure 2C, the production of NO was obviously inhibited by Ugonin M at the very low doses of 1.25 and 2.5 μg/mL.

### 2.3. Effect(s) of Ugonin M on LPS-Induced Acute Lung Injury

**Figure 3:**
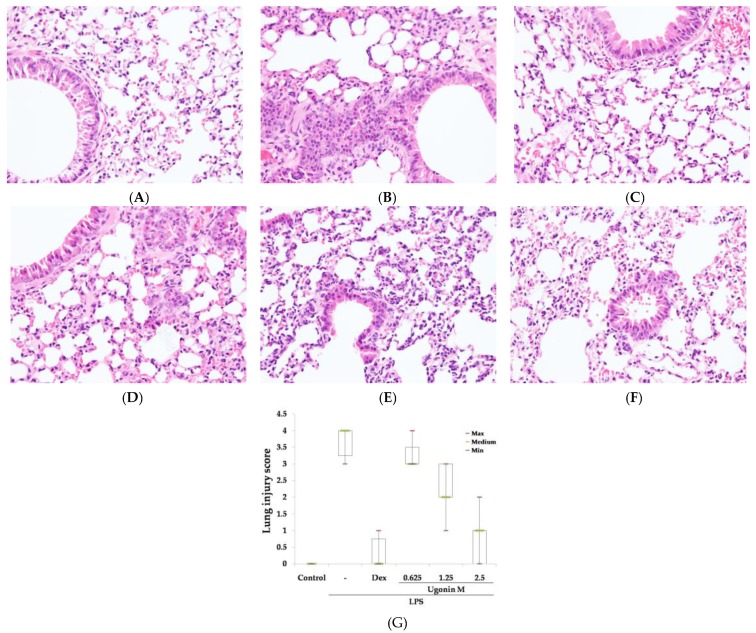
Effect of Ugonin M on lung histological changes in LPS-induced ALI mice: (**A**) Control; (**B**) LPS; (**C**) LPS + 10 mg/kg dexamethasone (Dex); (**D**) LPS + 0.625 mg/kg Ugonin M; (**E**) LPS + 1.25 mg/kg Ugonin M; and (**F**) LPS + 2.5 mg/kg Ugonin M. The infiltrating neutrophils were more abundant in (**B**) LPS group. The figure demonstrates a representative view (×400) from each group. (**G**) Lung injury score, of each group (*n* = 6) represents its non-parametric statistics.

As for the observation of pathological changes, hematoxylin and eosin (H&E) staining and the severity of lung injury were evaluated in this study. After being harvested from mice, lung tissue sections were soaked in formalin for two days before histological evaluation. As expected, the control group showed normal structures and no pathological changes in the lung tissues (Figure 3A). Figure 3B reveals the results of notable inflammatory neutrophils infiltration, interaalveolar septal thickening, interstitial and intraalveolar edema and patchy hemorrhage, and some collapsed alveoli. However, because of the pretreatments of dexamethasone (Dex) and Ugonin M, the pathological changes in lung tissues were relieved (Figure 3C–F). In addition, the severity of the lung injuries was scored by a blinded pathologist (Figure 3G).

### 2.4. Effect(s) of Ugonin M on Pulmonary Edema in Lung Tissue

**Figure 4:**
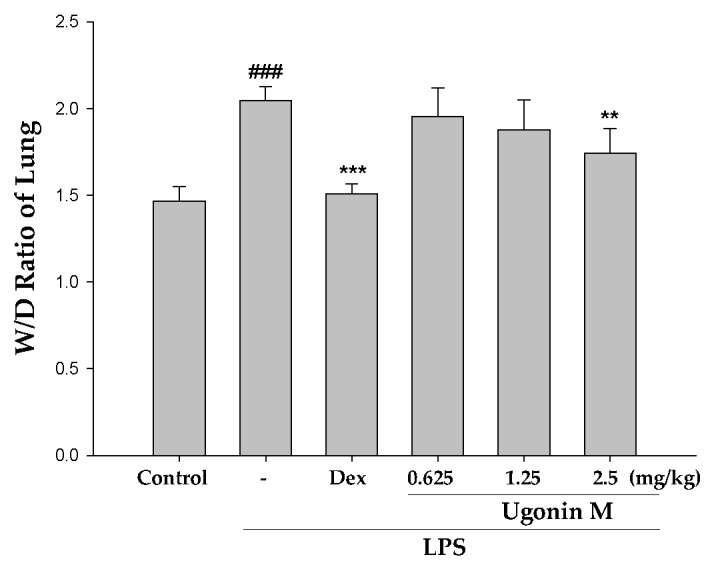
Effect(s) of Ugonin M on pulmonary edema. The right lower lungs were used to assess the W/D ratio of lungs. Each value represents the mean ± SD of six mice. ^###^ *p* < 0.001 was compared with a sample of the control group (one-way ANOVA followed by Scheffe’s multiple range tests). ** *p* < 0.01 and *** *p* < 0.001 were compared with the LPS-only group.

The ratio of the wet to dry weight (W/D ratio) of lungs is an important indicator to assess the severity of a pulmonary edema. As shown in Figure 4, with pretreatment of Ugonin M, the values of the W/D ratio were suppressed at 2.5 μg/mL (*p* < 0.01). In detail, the W/D ratio in the LPS group shows a remarkable difference compared with that of the control group.

### 2.5. Effect(s) of Ugonin M on Infiltrated Cellular Counts and Proteins Levels in BALF

**Figure 5:**
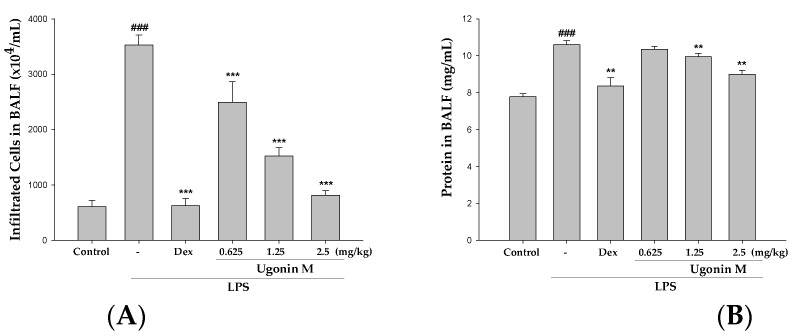
Effects of Ugonin M on: (**A**) infiltrated cellular counts; and (**B**) total proteins in BALF. Each value represents as the mean ± SD of six mice. ^###^ *p* < 0.001 was compared with a sample of the control group (One-way ANOVA followed by Scheffe’s multiple range tests). ** *p* < 0.01 and *** *p* < 0.001 were compared with the LPS-only group.

To further identify the anti-inflammatory feature of Ugonin M, the vascular permeability of the mice lungs was measured by determining the cellular counts and proteins content in the bronchoalveolar lavage fluid (BALF). As shown in Figure 5A,B, 6 h after LPS-induced ALI, the infiltrated cell numbers and protein concentration in the BALF significantly increased compared with the control group, whereas the mice pretreated with Ugonin M obviously demonstrated a trend toward lower levels.

### 2.6. Effect(s) of Ugonin M on NO, TNF-α, IL-6, and IL-1β Levels in BALF

**Figure 6:**
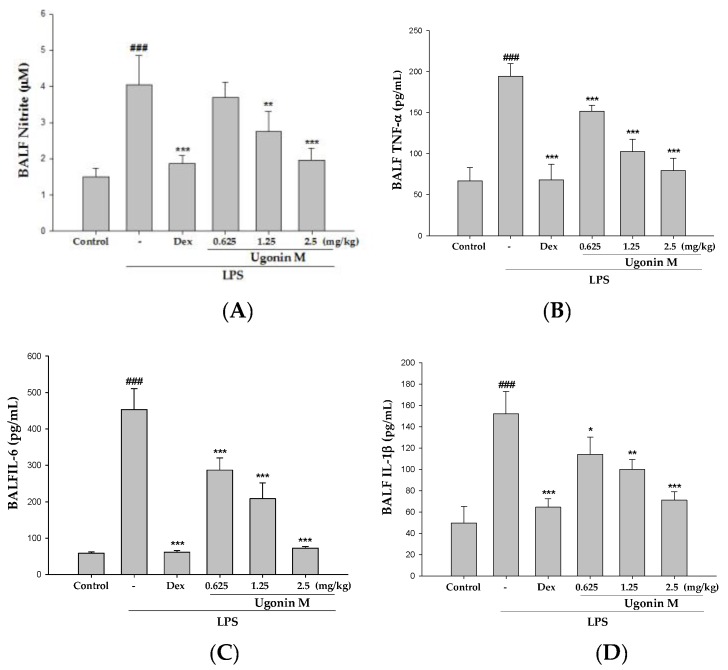
Effects of Ugonin M on: (**A**) NO; (**B**) TNF-α; (**C**) IL-6; and (**D**) IL-1β in BALF. Data represent the mean ± SD of six mice. ^###^ *p* < 0.001 was compared with a sample of the control group (One-way ANOVA followed by Scheffe’s multiple range tests). * *p* < 0.05, ** *p* < 0.01, and *** *p* < 0.001 were compared with the LPS-only group.

NO and the levels of pro-inflammatory cytokines, like TNF-α, IL-6, and IL-1β, in BALF at 6 h after the instillation of LPS were measured by ELISA. As shown in Figure 6, LPS challenge markedly increased the concentration of NO and pro-inflammatory cytokines as compared to the control group. Ugonin M administration significantly decreased the level of NO (Figure 6A), TNF-α (Figure 6B), IL-6 (Figure 6C), and IL-1β (Figure 6D). A positive control Dex group also significantly decreased the level of NO, TNF-α, IL-6, and IL-1β compared with the LPS group. The results below demonstrate that Ugonin M has the ability to reduce oxidative stress and inhibit the secretion of pro-inflammatory cytokines in LPS-induced ALI.

### 2.7. Effect(s) of Ugonin M on the Activity of MPO and Antioxidative Enzymes in Lung Tissue

**Figure 7:**
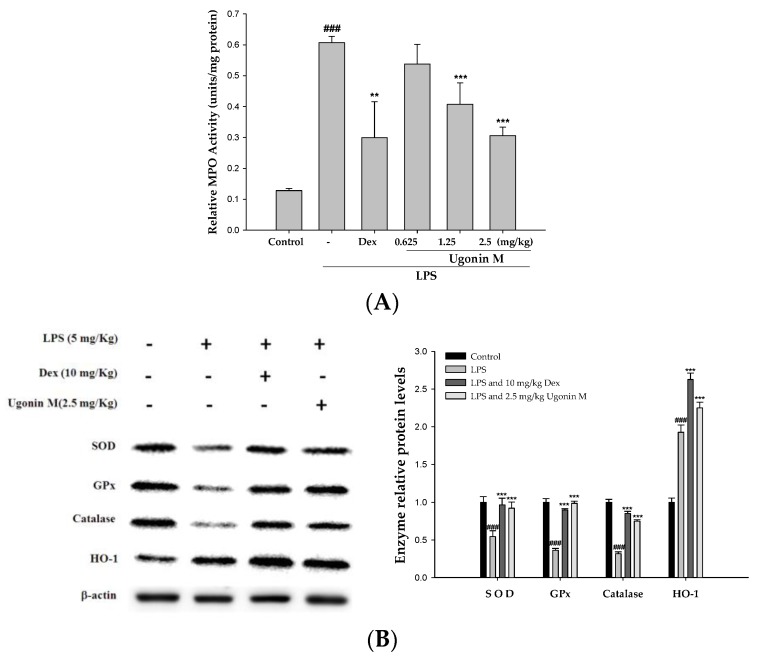
Effects of Ugonin M on: (**A**) MPO activity; and (**B**) antioxidative enzyme expression (SOD, GPx, catalase, and HO-1) in lungs from mice with ALI. Tissue homogenates were prepared and subjected to Western blotting. Data represent the mean ± S.D. for three different experiments performed in triplicate. ^###^ *p* < 0.001 was compared with a sample of the control group (One-way ANOVA followed by Scheffe’s multiple range tests). ** *p* < 0.01 and *** *p* < 0.001 were compared with the LPS-only group.

Myeloperoxidase (MPO) serves as an important index indicating neutrophils infiltration and polymorphonuclear neutrophil (PMN) burden, which have both been linked to the production of oxidative stress [21,22]. Moreover, although an increase in antioxidant defenses has been shown to occur in certain pulmonary diseases with increased oxidant burden, decreased antioxidant expression has been reported in other acute and chronic inflammatory respiratory diseases [23]. Superoxide dismutase (SOD), glutathione peroxidase (GPx), and catalase activities in the lung tissue were examined in this study to evaluate the antioxidative activity of Ugonin M. As expected with the results of the LPS group, the production of MPO in the lung tissues clearly increased after 6 h of the LPS challenge. In contrast to the LPS-only group, Ugonin M and Dex decreased the activity of MPO significantly (Figure 7A). In Figure 7B, because of pretreatments with Ugonin M, the expression of SOD, GPx, catalase, and HO-1 in LPS were obviously higher than that in the LPS-only group. These data suggest that Ugonin M has the ability to reduce LPS-induced oxidative stress.

### 2.8. Effect(s) of Ugonin M on iNOS and COX-2 Proteins Expression in Lung Tissue

**Figure 8:**
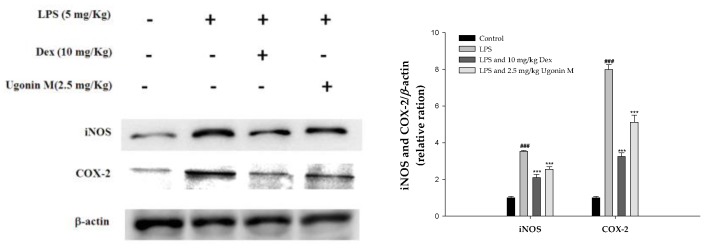
Effects of Ugonin M on iNOs and COX-2 protein expression in lung tissue. Six hours after LPS injection with or without Ugonin M pretreatment, the mice were exsanguinated and their lungs were removed. A representative Western blot from two separate experiments is shown, and its relative protein levels were calculated with reference to a LPS-only group. The data represent the mean ± SD for three different experiments performed in triplicate. ^###^ *p* < 0.001 was compared with a sample of the control group (One-way ANOVA followed by Scheffe’s multiple range tests). *** *p* < 0.001 was compared with the LPS-only group.

To assess the potential role of Ugonin M in LPS-induced ALI, we determined the level of cytokine proteins in the lung tissues of LPS-induced ALI mice using Western blot. As shown in Figure 8, the expression of the inducible isoform of NO synthase (iNOs) and COX-2 proteins were significantly inhibited with pretreatment of Ugonin M compared to the LPS-only group.

### 2.9. Effect(s) of Ugonin M on Activities of MAPK and NF-κB in Lung Tissue

**Figure 9:**
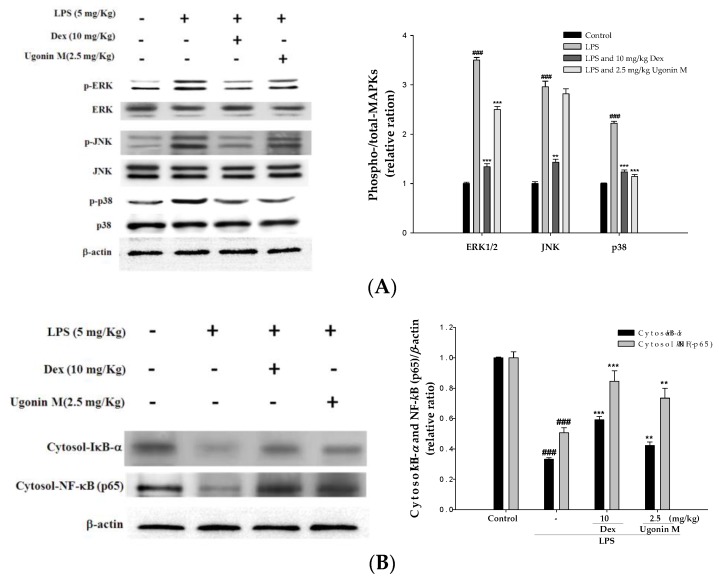

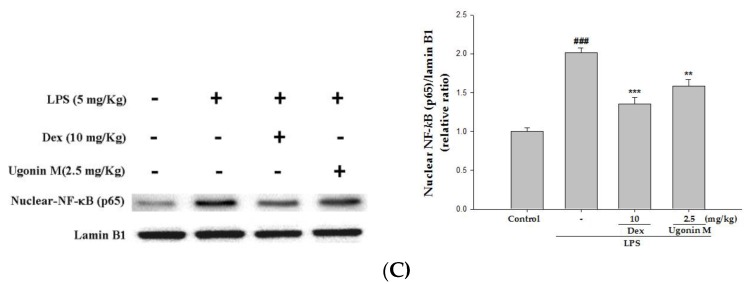
Effects of Ugonin M on LPS-induced: (**A**) MAPK phosphorylation and non-phosphorylation protein expressions; (**B**) cytosolic IκB-α and NF-κB; and (**C**) nuclear NF-κB concentrations in ALI mice. Tissue suspended were then prepared and subjected to Western blotting. Data represent the mean ± SD for three different experiments performed in triplicate. ^###^ *p* < 0.001 was compared with a sample of the control group (One-way ANOVA followed by Scheffe’s multiple range tests). ** *p* < 0.01 and *** *p* < 0.001 were compared with the LPS-only group.

Since MAPK (including three MAPK pathways: ERK, p38MAPK, and JNK) and NF-κB are critical pathways associated with an inflammatory response to LPS-induced ALI in mice, Western blot analysis was performed to evaluate the potential that Ugonin M inhibits the activity of MAPK. The results showed that LPS stimulation significantly increased MAPK phosphorylation, and Ugonin M significantly inhibited LPS-induced phosphorylation of ERK and p38MAPK (Figure 9A). 

To understand the effect(s) of Ugonin M on the degradation of IκB-α and the nuclear translocation of NF-κB, we evaluated the protein level of IκB-α and NF-κB translocation by Western blot assay.

Our findings show that the degradation of IκB-α and the translocation of NF-κB p65 increased significantly after LPS administration compared with the control group. Pretreatment with Ugonin M reduced the degradation of IκB-α and the translocation of NF-κB inducing by LPS (Figure 9B,C). These data indicate that Ugonin M relieved LPS-induced ALI by inhibiting NF-κB activation through the degradation of NF-κB and IκB-α as well as ERK and p38MAPK active phosphorylation pathways.

### 2.10. Effect(s) of Ugonin M on TLR4 Expression in Lung Tissues

**Figure 10:**
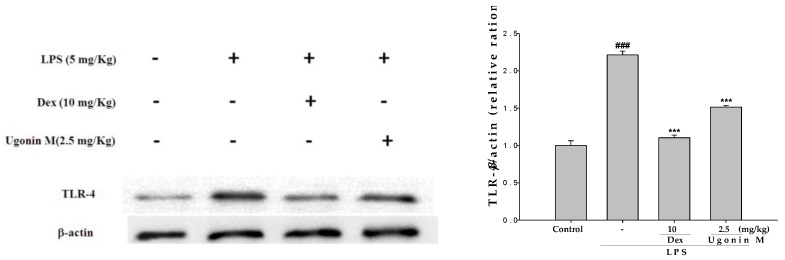
Effect(s) of Ugonin M on LPS-induced TLR-4 expression in lung tissues. The fold change in TLR-4 expression between the pretreated and the control groups was calculated. Data represent the mean ± SD for three different experiments performed in triplicate. ^###^ *p* < 0.001 was compared with sample of control group (One-way ANOVA followed by Scheffe’s multiple range tests). *** *p* < 0.001 was compared with the LPS-only group.

To examine whether the inhibition of pro-inflammatory molecules by Ugonin M is associated with down-regulation of TLR4, the effect of Ugonin M on TLR4 expression was analyzed by Western blotting. We found that LPS stimulation exhibited an important result as shown in Figure 10: Ugonin M inhibits the LPS-induced up-regulation of TLR4 expression, significantly. This suggests that Ugonin M, with its down-regulation of TLR4 expression, has the ability to reduce LPS-induced inflammatory damage.

## 3. Discussion

As shown in Figure 1, Ugonin M is one of the major components of *H. zeylanica*, a famous anti-inflammatory herbal medicine in traditional Chinese medicine [9]. We isolated it from *H. zeylanica* in accordance with the method devised by Huang et al. [14] with only minor modification.

To evaluate the appropriate and effective dose of Ugonin M, we examined the cytotoxicity and the ability of NO inhibition of Ugonin M on LPS-stimulated RAW264.7 cells. The results were that Ugonin M at the dose of 2.5 mg/kg was not cytotoxic and clearly down-regulated NO production in an in vitro experiment (Figure 2). These results told us that Ugonin M has a potential protective effect in LPS-induced ALI in mice.

In the in vivo experiment, the results in Figure 3 showed that pretreatment with different concentrations of Ugonin M and Dex significantly ameliorated the pulmonary tissue injury when compared to the LPS-only group. A lung edema is one of the critical distinguishing features of ALI. Thus, the lung W/D ratio and the concentrations of protein and infiltrated cells in BALF were also investigated. Our results demonstrated that, compared to the LPS-only group, the lung W/D ratio was clearly reduced in a dose-dependent manner, 6 h after LPS injection with or without Ugonin M pretreatment (Figure 4).

In ALI, a complex network of cytokines and chemokines, such as IL-6, IL-1β, among others, mediates the inflammatory response. In mice with LPS-initiated ALI, IL-6 in the plasma and the BALF of humans have been found to be related to an increased risk of developing ALI [24]. We analyzed several inflammatory parameters, including the numbers of cellular counts, proteins, and pro-inflammatory cytokines involving IL-6, IL-1β, and TNF-α production in BALF to estimate the vascular permeability of the lung tissue studied and the ability of Ugonin M to inhibit the production of pro-inflammatory cytokines. The key results (Figure 5 and Figure 6) from our study were that Ugonin M improved the vascular permeability alterations and pro-inflammatory cytokines in a dose dependent manner, and that the efficacy between the high dose group and the positive control group was similar.

An acute inflammatory process could be associated with neutrophil-derived active oxygen species and free radicals, such as hydrogen peroxide, superoxide, nitric oxide (NO), and cytokines. The expression of iNOS has been proposed to be an important mediator of inflammation [25]. Neutrophils are considered one of the first responders of the innate immune response. Measurement of neutrophil infiltration into tissues is one way to gauge the severity of infection, inflammation, and tissue damage. MPO is found in the primary granules of neutrophils and is an effective measure of neutrophil infiltration into tissues [26]. MPO, a heme protein secreted by activated leukocytes, catalyzes the oxidation of tyrosine and nitrite to form a tyrosyl radical and nitrogen dioxide (‧NO_2_), individually, reactive intermediates capable of initiating lipid oxidation and generating bioactive eicosanoids during inflammation [27]. SOD, GPx, catalase, and HO-1 are inducible defense enzymes against oxidative stress. HO-1 is a kind of antioxidative protein that catabolizes Heme and subsequently ameliorates symptoms of ALI through the inhibition of NF-κB phosphorylation [5]. The results (Figure 7) of our study demonstrate that Ugonin M and Dex reduced lung tissue MPO formation. In addition, there were significant increases in SOD, GPx, catalase, and HO-1 activities with Ugonin M treatment. Thus, Ugonin M could effectively scavenge free radicals, inhibit lipid peroxidation, and protects tissues from inflammatory damage [25]. In addition, Ugonin M at doses of 2.5 mg/kg significantly inhibited LPS-induced NO production and iNOS and COX-2 protein expressions in BALF (Figure 6A) or in lung tissue (Figure 8). Generally, flavonoid compounds effectively scavenge free radicals and inhibit NO production independent of their antioxidant properties. According to the above results, Ugonin M has been shown to be an endogenous factor in reducing oxidative stress. These findings suggest Ugonin M could be a potential alternative for the treatment of LPS-induced ALI.

To further understand the molecular mechanism(s) of the inhibitory effect of Ugonin M on the production of cytokine, we evaluated the performance of Ugonin M on the concentration of IκB-α and NF-κB in cytosol. NF-κB is one of the vital nuclear transcription factors that act as regulators in immune and inflammatory processes by the regulation of pro-inflammatory cytokines and chemokines [21,28]. In the absence of stimuli, NF-κB is sequestered in the cytoplasm, where it binds to IκB-α and remains inactive. Once activated, NF-κB p65 dissociates from its inhibitory proteins IκB and translocates from the cytoplasm to the nucleus where it triggers the transcription of specific genes such as TNF-α, IL-1β, and IL-6. In addition, studies have shown that NF-κB/MAPK signaling pathways play a pivotal role in the pathogenesis of LPS-induced ALI [29,30]. We found that pretreatment with Ugonin M could prevent the nuclear translocation of NF-*κ*B and downregulate the phosphorylation of ERK and p38MAPK in lung tissue, suggesting that Ugonin M has protective effects on LPS-induced ALI through regulating the NF-κB and MAPK pathways (Figure 9).

TLR4, serving as a critical pattern recognition receptor of host immune responses and an indispensable upstream sensor for LPS, could mediate the MyD88-dependent and MyD88-independent (TRAM/TRIF-mediated) pathways to adjusts the activation of NF-κB and MAPK, affect the release of related inflammatory cytokines and thus regulate the injury of lung tissue mediated by cytokines [8,21,31,32,33]. In this study, the result in Figure 10 revealed that the promoted expressions of TLR4 by LPS challenge were obviously downregulated with the pretreatment of Ugonin M, which conformed with the number changes of IκBα, NF-κB p65, MAPK, and other pro-inflammatory cytokines in ALI lung tissue or BALF.

In summary, this is the first study showing that Ugonin M, the major component of *H. zeylanica*, suppresses LPS-induced lung injuries. We have demonstrated that Ugonim M not only suppresses the production of pro-inflammatory molecules, as such, TNF-α, IL-6, and IL-1β, it also inhibit NO production and iNOS and COX-2 protein expression through TLR4-mediated MAPK and NF-κB signaling pathways. In addition to its potential in increasing SOD, GPx, and catalase production, it is able to induce the HO-1 protein expression in order to defence against oxidative damage in LPS-induced lung injuries. These findings provide strong evidence that Ugonin M may be a potential component as an anti-inflammatory agent in ALI. Our study may also support the notion that Ugonin M is one of the major components of *H. zeylanica* in the treatment of inflammatory disorders. As we have discovered, Ugonin M exerts an anti-inflammatory effect in rodents.

## 4. Materials and Methods

### 4.1. Isolation and Determination of Ugonin M from *H. zeylanica*

The root and rhizome of *H. zeylanica* were purchased from a local herbal medicine store in Taichung, Taiwan, and authenticated by Dr. Yuan-Shiun, Chang. A voucher specimen (CMU-CMR-HZ-103001) was deposited at the Department of Chinese Pharmaceutical Sciences and Chinese Medicine Resources for further reference. The dried material (5 kg) was extracted with ethanol (10 L, four times) at room temperature. The extract solution was filtered and filtrate was concentrated by evaporation under reduced pressure to produce an ethanol extract (151.22 g, yield rate: 3.02%). The extract was suspended in distilled water, and the aqueous suspension was partitioned with *n*-hexane, ethyl acetate, and *n*-butanol, sequentially. The ethyl acetate layer was evaporated until dry, and the residue was chromatographed on silica gel with *n*-hexane:acetone (7:3) to elute a crude fraction that included Ugonin M. This fraction was purified by silica gel column and HPLC to obtain 102 mg of Ugonin M (Figure 2A). The structure of Ugonin M (yellow powder, C_25_H_24_O_7_) was identified by detailed analysis of 1D-NMR spectroscopic data: 1H-NMR (CDCl_3_, 500 MHz) δH: 6.80 (1H, s, H-5′), 6.34 (4H, d, *J* = 2.1 Hz, H-8), 6.19 (1H, d, *J* = 2.1 Hz, H-6), 5.08 (1H, brs, H-12), 3.23 (1H, dd, *J* = 12.5, 5.8 Hz, H-9a), 2.91 (1H, dd, *J* = 12.5, 10.3 Hz, H-9b), 2.20 (1H, dd, *J* = 10.3, 5.8 Hz, H-10), 2.10 (1H, m, H-13a),2.00 (1H, m, H-13b), 1.92 (1H, m, H-14a),1.24 (1H, m, H-14b), 1.07 (3H, s, H-18), 0.95 (3H, s, H-17), 0.88 (3H, s, H-16). The result was confirmed by comparison with previously published literature [15].

### 4.2. HPLC Analysis of *H. zeylanica*

HPLC analyses were performed on a Waters 2695 HPLC system equipped with a Waters 2998 photodiode array detector (PDA, Waters Corporation, Milford, MA, USA), Waters e2695 separations module and column heater module. A Merck (Billerica, MA, USA) Purospher^®^ STAR Shield RP 18 column (250 mm × 4.6 mm, 5 μm) was used. The mobile phase consisted of methanol (A) and phosphoric acid in water (0.1%, *v*/*v*) (B). The optimized elution conditions were as follow: 5–45 min, 70%–80% A; 50–55 min, 80%–70% A. The flow rate was 1 mL/min and the injection volume was 10 μL. UV spectra were acquired from 190 nm to 400 nm. The autosampler and column compartment were maintained at 25 °C and 35 °C, respectively.

### 4.3. Cell Culture

A murine macrophage cell line RAW 264.7 (BCRC No. 60001) was purchased from the Bioresources Collection and Research Center (BCRC) of the Food Industry Research and Development Institute (Hsinchu, Taiwan). Cells were cultured in plastic dishes containing Dulbecco’s Modified Eagle Medium (DMEM, Sigma, St. Louis, MO, USA) supplemented with 10% fetal bovine serum (FBS, Sigma, St. Louis, MO, USA) in a humidified atmosphere of 5% CO_2_ and 95% air at 37 °C and subcultured every three days at a dilution of 1:5 using 0.05% trypsin-0.02% EDTA in Ca^2+^-, Mg^2+^-free phosphate-buffered saline (DPBS) [34].

### 4.4. Cytotoxicity and the Measurement of Nitric Oxide

This preliminary experiment was made up of two parts. In part one, we inspected the viability of RAW 264.7 cells; in part two, and cell medium was examined for production of NO.

RAW 264.7 cells (5 × 10^4^ per well) were seeded in 96-well plates containing DMEM supplemented with 10% FBS for 24 h to become nearly confluent. On the second day, the cells were pretreated with the indicated concentrations of Ugonin M one hour before treatment with LPS (100 ng/mL) at 37 °C for 24 h. The media were removed and stored for the following NO experiment, while the cells were incubated with 100 μL of 0.5 mg/mL MTT in a CO_2_ incubator (5% CO_2_ in air) for 6 h at 37 °C after testing for cell viability. The medium was discarded, followed by the addition of 100 μL 0.04N HCl/isopropanol. The optical density (OD) at 570 nm was measured using a microplate reader. The above steps were repeated three times for each concentration.

The nitrite level in cultured media, which reflects intracellular NO synthase activity, was based on the Griess reaction [25]. One hundred microliters of Griess reagent (1% sulfanilamide, 0.1% naphthyl ethylenediamine dihydrochloride, and 5% phosphoric acid) was added to each sample medium and incubated at room temperature for 10 min. Absorbance was read at 540 nm. Nitrite levels in the samples were calculated from a standard curve with known concentrations of sodium nitrite.

### 4.5. Animals

Seventy-two male imprinting control region (ICR) mice, 6 weeks old, were obtained from BioLASCO Co., Ltd. (Taipei, Taiwan). The animals were kept in plexiglass cages at a constant temperature of 22 ± 1 °C, relative humidity 55% ± 5% and with 12 h dark-light cycles for at least one week before experiment. They were given food and water *ad libitum*. Our animal studies were conducted according to the regulations of Instituted Animal Ethics Committee, and the animal use protocol (Protocol NO.: 104–234; date of approval: 2015.02.03) was approved by the Institutional Animal Care and Use Committee, China Medicine University. After an adaptation period of seven days, male ICR mice were randomly divided into six groups (*n* = 12). Mice in the normal and negative control group were administrated with sterile saline (intraperitoneal; *i.p.*). The other four groups included a positive control (Dexamethasone), three Ugonin M pretreatment groups (0.625, 1.25, and 2.5 mg/kg, respectively) were prepared in a solvent of sterile saline in 0.5% carboxymethyl cellulose.

### 4.6. Model of LPS Induced ALI

ALI was induced by LPS (*E. coli* LPS serotype O55:B5, Sigma, St. Louis, MO, USA) via intratracheal injection [35]. In brief, mice were anesthetized with a mixed reagent of 10 μL/g *i.p.*, urethane (0.6 g/mL) and chloral hydrate (0.4 g/mL), then followed by Dex (10 mg/kg) or Ugonin M *i.p.* injection, with individual doses as a pretreatment. One hour after above pretreatment, we instilled LPS (5 mg/kg) intratracheally to induce ALI. The mice were then placed in a vertical position and rotated for 1 min to disperse the instillation in the lungs. Six hours later, the mice were exsanguinated. Half of each group was taken for analysis of BALF, and the rest were used for the analysis of inflamed proteins, slicing, and edema.

### 4.7. Histological Examination

The left lower lung from each mouse was fixed in 10% formalin, embedded in paraffin, cut into 5 mm sections, and then treated with an H&E stain for histological examination. Lung injury score was observed and scored by a blinded pathologist and the degree of lesions was from 1 to 5 depending on severity: 1 = minimal (<1%); 2 = slight (1%–25%); 3 = moderate (26%–50%); 4 = moderate/severe (51%–75%); and 5 = severe/high (76%–100%).

### 4.8. Lung Wet to Dry (W/D) Weight Ratio

The lung tissues were excised and weighed, immediately, and then dried at 60 °C for 72 h in an oven to acquire their dry weight. The W/D ratio was then calculated to assess the severity of the pulmonary edemas.

### 4.9. Bronchoalveolar Lavage Fluid (BALF), Total Cell Count and Protein Analysis

Based on a previous report [36], the upper part of the tracheas was cannulated and lavaged three times with 600 μL PBS (pH 7.2) each time to obtain BALF. BALF was centrifuged at 700× *g* at 4 °C for 5 min, and the supernatant was removed for the following cytokines assay. The sedimented cells were re-suspended in 2 mL PBS, of which 1 mL was used to determine the number of infiltrating leukocytes by cytometer and the other was added with a RIPA solution (radioimmuno-precipitation assay buffer) and then centrifuged again to obtain the supernatant so as to detect total protein content by Bradford assay.

### 4.10. TNF-α, IL-6, and IL-1β Cytokines in BALF

The concentrations of the pro-inflammatory cytokines levels of TNF-α, IL-6, and IL-1β in BALF were measured by a commercially available enzyme-linked immunosorbent assay kit (Minneapolis, MN, USA) in accordance with the manufacturer’s instructions.

### 4.11. Myeloperoxidase (MPO) Activity Assay

After BALF collection, the left upper lobe of the lungs was removed, washed, and kept at −80 °C. The steps were conducted according to the method of Huang et al. [37]. After weighing, the lungs were homogenized at 12,000 × *g* at 4 °C for 15 min and re-suspended in a 50 mM K_3_PO_4_ buffer (PH 6.0) containing 0.19 mg/mL of *o*-dianisidine chloride and 0.0005% H_2_O_2_ as a substrate. Oxidized *o*-dianisidine was measured by spectrohpotometry (OD _460 nm_). MPO values were reported as changes in optical density per minute per gram of wet lung.

### 4.12. Western Blot Analysis of the Lung Tissues

PBS and RIPA were added to the lung tissues before grinding. The extract was then centrifuged at 12,000× *g* at 4 °C for 15 min to obtain the supernatant. Bovine serum albumin (BSA) was used as a protein standard to calculate the equal total cellular protein amounts. Fifty micrograms of Protein samples were resolved by denaturing 10% sodium dodecyl sulfate-polyacrylamide gel electrophoresis (SDS-PAGE) using standard methods, and then were transferred onto PVDF membranes by electroblotting and blocking with 1% BSA (overnight). The membranes were incubated with an appropriate dilution of specific primary antibodies (SOD, GPx, catalase, HO-1, iNOS, COX-2, cytosolic IκBα, cytosolic NF-κB, and TLR-4), for phosphorylated and non-phosphorylated forms of ERK, p38MAPK, JNK at 4 °C, washed three times with PBST, and incubated for 1 h at 37 °C with horseradish peroxidase-conjugated secondary antibodies (overnight). The membranes were washed three times before being checked for immuno-reactive proteins by enhanced chemiluminescence (ECL) using hyperfilm and an ECL reagent. Band intensity on scanned films was quantified by using Image J Software (NIH, Bethesda, MD, USA) and represented as relative intensity compared with the control group.

### 4.13. Statistical Analysis

Unless otherwise stated, experiment results were performed at least three times independently. The data were reported as the means ± standard deviation (S.D.), and statistical comparisons between the groups were carried out by one-way ANOVA, followed by a Scheffe’s multiple range test. The criterion for statistical significance was set at a *p*-value of less than 0.05. Lung injury score is presented by non-parametric statistics.
